# Precentral gyrus functional connectivity signatures of autism

**DOI:** 10.3389/fnsys.2014.00080

**Published:** 2014-05-14

**Authors:** Mary Beth Nebel, Ani Eloyan, Anita D. Barber, Stewart H. Mostofsky

**Affiliations:** ^1^Department of Neurology, Johns Hopkins School of MedicineBaltimore, MD, USA; ^2^Laboratory for Neurocognitive and Imaging Research, Kennedy Krieger InstituteBaltimore, MD, USA; ^3^Department of Biostatistics, Johns Hopkins Bloomberg School of Public HealthBaltimore, MD, USA; ^4^Department of Psychiatry, Johns Hopkins School of MedicineBaltimore, MD, USA

**Keywords:** autism, functional connectivity, motor cortex, multi-center studies, logistic regression, resting state fMRI

## Abstract

Motor impairments are prevalent in children with autism spectrum disorders (ASD) and are perhaps the earliest symptoms to develop. In addition, motor skills relate to the communicative/social deficits at the core of ASD diagnosis, and these behavioral deficits may reflect abnormal connectivity within brain networks underlying motor control and learning. Despite the fact that motor abnormalities in ASD are well-characterized, there remains a fundamental disconnect between the complexity of the clinical presentation of ASD and the underlying neurobiological mechanisms. In this study, we examined connectivity within and between functional subregions of a key component of the motor control network, the precentral gyrus, using resting state functional Magnetic Resonance Imaging data collected from a large, heterogeneous sample of individuals with ASD as well as neurotypical controls. We found that the strength of connectivity within and between distinct functional subregions of the precentral gyrus was related to ASD diagnosis and to the severity of ASD traits. In particular, connectivity involving the dorsomedial (lower limb/trunk) subregion was abnormal in ASD individuals as predicted by models using a dichotomous variable coding for the presence of ASD, as well as models using symptom severity ratings. These findings provide further support for a link between motor and social/communicative abilities in ASD.

## Introduction

Defined by impairments in social reciprocity and communication, as well as the presence of restricted, repetitive behaviors, Autism Spectrum Disorder (ASD) is a pervasive diagnosis that emerges early in life and is generally associated with life-long disability (Howlin et al., 2004). In addition to these defining features, a growing body of evidence suggests that atypical motor functioning is also prevalent in ASD. Prospective studies have revealed that motor abnormalities associated with ASD are observable in infancy (Baranek, 1999; Landa and Garrett-Mayer, 2006; Brian et al., 2008), and measurable motor impairments persist throughout childhood and into adulthood (Hallett et al., 1993; Jansiewicz et al., 2006; Freitag et al., 2007). Motor difficulties experienced by individuals with ASD may also be heritable and part of the broader ASD phenotype. Early motor delays are more common in infant siblings of children with ASD who are at high risk for ASD than in low-risk infants without ASD siblings, and these early motor delays predict future communication delays in high risk children (Bhat et al., 2012).

Motor impairments experienced by individuals with ASD also appear to be linked with the social and communicative impairments that define the diagnosis. The mastery of motor skills is critical for the development of a wide range of abilities related to social reciprocity including interacting with others (Clearfield, 2011) and mental imagery (Williams et al., 2008). It is likely that mechanisms involved in the abnormal development of motor skills in ASD also contribute to the impaired development of social and communicative abilities in these individuals (Mostofsky et al., 2000; Ullman, 2004). The severity of motor impairments experienced by children with ASD relates to the severity of the communicative/social deficits at the core of their diagnosis (Dziuk et al., 2007; Dowell et al., 2009), and better early motor control has been shown to be related to decreased severity of ASD in later life (Sutera et al., 2007). Considered together, these converging lines of evidence suggest that a purely cognitive explanation for ASD is shortsighted and underscore the need for systematic examination of the neural underpinnings of motor development in individuals with ASD.

Evidence suggests that motor impairments experienced by individuals with ASD may reflect structural and functional connectivity abnormalities within brain networks underlying motor control and learning. Increased radiate white matter volume within primary motor cortex (Mostofsky et al., 2007) and deformations in basal ganglia shape have been shown to be correlated with motor dysfunction in children with ASD (Qiu et al., 2010). Functional Magnetic Resonance Imaging (fMRI) evidence has indicated that adults with ASD exhibit more spatially diffuse activations in motor-related regions of the cerebellum compared to neurotypical controls when engaged in simple motor tasks (Allen et al., 2004); children with ASD demonstrate reduced functional connectivity (FC) compared to typically developing children throughout the motor control network during finger sequencing (Mostofsky et al., 2009). In addition to these structural and task-related findings, resting state fMRI investigations have reported reduced interhemispheric connectivity between right and left somatomotor cortex (Anderson et al., 2011), as well as potentially delayed functional segregation of the precentral gyrus (PCG) in children with ASD compared to their typically developing (Nebel et al., 2014).

Although motor abnormalities in ASD are well-characterized, there remains a fundamental disconnect between the complexity of the clinical presentation of ASD and the underlying neurobiological mechanisms. Relatively few studies have directly linked abnormal connectivity in individuals with ASD to specific symptoms (Weng et al., 2010; Dinstein et al., 2011; Uddin et al., 2013). However, there is a growing literature demonstrating that inter-individual variability in functional connectivity is systematically related to various measures of behavioral variability in neurotypical controls including motor performance (Barber et al., 2012), sequence learning (Stillman et al., 2013), and reading ability (Zhang et al., 2013). One substantial obstacle contributing to the disconnect between connectivity metrics and ASD symptom severity has been the small sample sizes analyzed relative to the heterogeneity of ASD (Nielsen et al., 2013) and the noisiness of connectivity measures.

The public release of the Autism Brain Imaging Data Exchange (ABIDE) presents an opportunity to investigate functional connectivity abnormalities associated with ASD in a large, heterogeneous dataset (Di Martino et al., 2013). ABIDE compiled 1112 anatomical and resting state fMRI scans from multiple international imaging centers with the intent of creating a dataset roughly balanced in terms of the numbers of subjects with ASD (539) and neurotypical controls (573), and in terms of participant age across groups. However, as was illustrated by the ADHD-200 Global Prediction competition, tractability becomes a serious concern with such large-scale datasets (Eloyan et al., 2012; Sidhu et al., 2012). Even when collected at one site using consistent subject recruitment strategies and imaging parameters, the fluctuations captured by resting state fMRI represent noisy signals from a large number of sources, and the majority of the variance in the imaging data will not be useful for understanding the neurobiology of ASD. The trick to harnessing the potential power of this imaging resource will be to use the data intelligently so that variability relevant to ASD is not overpowered by the billions of unnecessary numbers included in the dataset (Caffo et al., 2011). Several groups have proposed segmenting the brain prior to functional connectivity analysis to reduce the dimensionality of the data and to enable rapid calculation of inter-parcel FC signatures for individual subjects (Wang et al., 2009; Faria et al., 2012). Motivated by these potentially scalable methods to investigate brain organization as well as by the evidence of abnormal functional segregation of the PCG in ASD, this study set out to test two main hypotheses: (1) that functional connectivity within the PCG will be abnormal in individuals with ASD compared to neurotypical control participants due to reduced functional segregation within the PCG and (2) that the strength of connectivity between subregions of the PCG is associated with the severity of autistic traits using a large, heterogeneous sample of resting state data.

## Materials and methods

### Data inclusion

**Figure 1:**
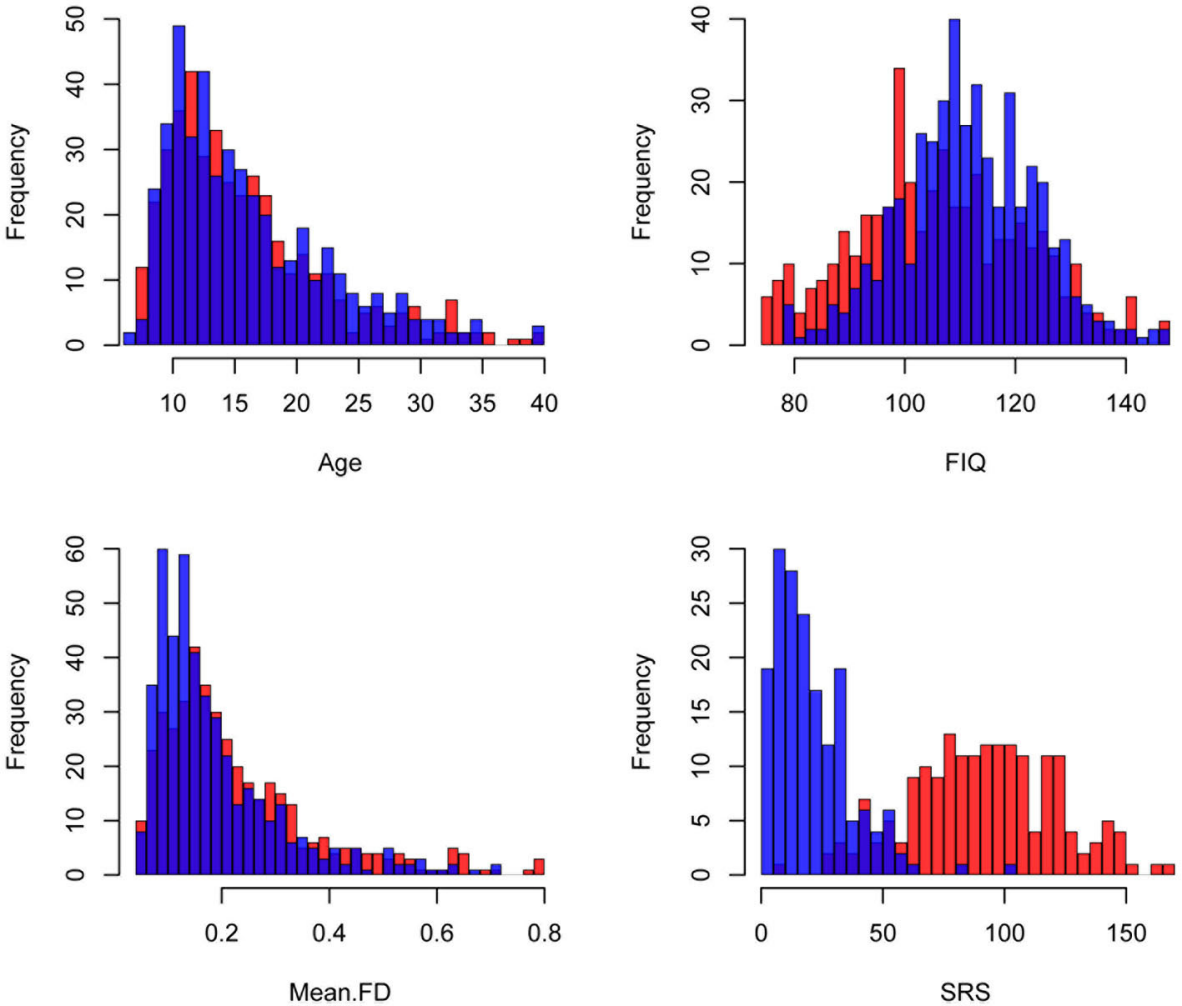
**Demographic information**. Histograms of the demographic variables included in these analyses are colored by disease status. Red: Autism Spectrum Disorder (ASD), Blue: Controls. FIQ: Full Scale Intelligence Quotient, Mean FD: mean Framewise Displacement, SRS: total Social Responsiveness Scale score.

A resting state fMRI scan and an anatomical image were provided for each participant in the ABIDE dataset. Data from an additional 58 children collected at Kennedy Krieger Institute after the ABIDE release were also included in this analysis. Details of recruitment, informed consent, acquisition, and other site-specific protocols for all ABIDE contributors are available online (fcon_1000.projects.nitrc.org/indi/abide/). All protocols were in compliance with the policies of site-specific institutional review boards and of HIPAA regulations regarding the anonymization of shared information. Each site established different criteria for diagnosing patients with autism and for ascertaining typical development; however, the majority of the sites used the Autism Diagnostic Observation Schedule (ADOS) (Lord et al., 2000) and the Autism Diagnostic Interview-Revised (Lord et al., 1994). The following inclusion criteria were used to reduce non-informative variance in our analyses: (1) male, as females represented only 10% of the aggregate sample; (2) with an estimated Full Scale Intelligence Quotient (FIQ) greater than or equal to 75; (3) between the ages of 6 and 40 years old, as only 2% of the sample represented individuals older than 40; (4) whose between-volume head movement as summarized by mean framewise displacement (FD) (Power et al., 2012) was within 2 standard deviations of the mean for the sample, and (5) whose images were successfully registered to standard space. These criteria yielded data from 868 participants (450 ASD, 418 controls). The distributions of age, FIQ, and mean FD for the subjects in each group who satisfied these criteria across all sites are illustrated in Figure 1. The distribution of overall autism trait severity as measured by the total raw Social Responsiveness Scale score is also displayed in Figure 1 for the sites that provided this information. Table 1 breaks down these demographic parameters by data-contributing site.

**Table 1:** **Demographic information by data-contributing site**.

	**N**	**Diagnosis**		**Age**			**Full IQ**		
		**NT**	**ASD**	**Mean**	**Median**	**SD**	**Mean**	**Median**	**SD**
Total	868	450	418	16.1	14.4	6.57	109.4	109	14.33
CALTECH	23	11	12	24.9	22.9	6.72	111.4	111	12.2
CMU	20	9	11	25.95	25	5.12	111.8	109	10.13
KKI	89	50	39	10.27	10.38	1.27	111.6	112	15.15
LEUVEN1	29	15	14	22.59	22	3.55	112.2	109	13.03
LEUVEN2	0	0	0	0	0	0	0	0	0
MAX_MUN	41	26	15	21.98	23	8.39	109.7	110	11.29
NYU	147	79	68	15.03	13.20	6.41	111	109	14.76
OHSU	25	15	10	10.41	10.22	1.51	113.3	115.2	14.9
OLIN	26	13	13	16.65	17	3.45	115	116.5	15.76
PITT	47	21	26	19.63	17.36	6.86	109.9	110	12.28
SBL	3	0	3	31.67	33	4.16	105.3	96	17.04
SDSU	28	16	12	14.78	14.92	1.55	112.2	112	13.14
STANFORD	32	16	16	10.07	9.62	1.62	113.1	114	16.18
TRINITY	46	24	22	17.28	16.93	3.58	110.4	113	12.52
UCLA1	63	27	36	13.34	13.38	2.27	103.6	105	11.52
UCLA2	22	12	10	12.42	12.4	1.6	104.3	103.5	14.16
UM1	68	35	33	13.4	12.8	2.78	105.8	107	13.73
UM2	32	20	12	16.06	15.3	3.44	112.5	113.2	11.08
USM	91	42	49	21.8	20.18	6.71	107.4	108	16.07
YALE	36	19	17	12.23	12.29	2.83	102.6	101	17.68

ASD, Autism Spectrum Disorder; Caltech, California Institute of Technology; CMU, Carnegie Melon University; FD, Framewise Displacement; KKI, Kennedy Krieger Institute; LEUV, University of Leuven; MAXMUN, Ludwig Maximilians University of Munich; N, number of subjects; NT, Neurotypical Control; NYU, New York University Langone Medical Center; OHSU, Oregon Health and Science University; OLIN, Olin Institute of Living at Hartford Hospital; PITT, University of Pittsburgh School of Medicine; SBL, Social Brain Lab Netherlands Institue for Neuroscience and the Unversity Medical Center Groningen; SD, standard deviation; SDSU, San Diego State University; STAN, Stanford University; TRINITY, Trinity Centre for Health Sciences; UCLA, University of California at Los Angeles; UM, University of Michigan; USM, University of Utah School of Medicine.

### Image processing

Image processing was performed using SPM12b (Wellcome Department of Imaging Neuroscience) and custom MATLAB (The Mathworks, Inc.) scripts run on the Joint High Performance Computing Exchange (http://jhpce.jhu.edu). The volumes corresponding to the first 10 s of each resting state scan were discarded to allow for magnetization stabilization. The resting state data were then slice-time adjusted using the slice that was acquired at the middle of the repetition time (which varied by site). Rigid body realignment parameters were estimated with respect to the first (stabilized) functional volume of the scan and used to calculate mean FD. An iterative process was used to coregister and normalize the anatomical and functional images. SPM12b's segmentation tool was first used to generate a bias-corrected version of the anatomical image, which was coregistered to the first (stabilized) functional volume. The segmentation tool was reapplied to the bias-corrected anatomical image and the resulting deformation used to transform the bias-corrected image into Montreal Neurological Institute (MNI) template space. This non-linear spatial transformation was then applied to the functional data along with the estimated rigid body realignment parameters and resulted in 2-mm isotropic voxels. To insure the consistency of spatial normalization across participants, we visually spot-checked registration to MNI space at a series of landmarks representing the anterior, posterior, inferior, superior, and lateral extremes of the brain in SPM's T1 template.

Each resting state scan was temporally detrended on a voxelwise basis to remove linear trends. An aCompCor strategy was used to estimate spatially coherent noise components from tissues not expected to exhibit Blood Oxygenation Level Dependent signals (Behzadi et al., 2007) and remove them from the data. Tissue probability maps generated during segmentation for white matter (WM) and cerebral spinal fluid-filled spaces (CSF) were restricted using a 99% probability threshold. The resulting WM mask was eroded using MATLAB's imerode function to reduce the risk of including signals of interest from gray matter. The CSF mask was constrained to areas within the ALVIN mask of the ventricles (Kempton et al., 2011). Principal components (PCs) explaining 50% of the variance in WM and 50% of the variance in CSF were then regressed from the resting state data along with linearly detrended versions of the six rigid body realignment parameters and the first derivative of each linearly detrended realignment parameter (computed by backward differences). During our first attempt to process the data, we hard-coded the number of PCs included from WM and CSF to 5 each and found that motion effects persisted in our data. Setting the number of PCs included in the nuisance regression to reflect the variance explained from each of these regions appeared to better attenuate the persistent effects of motion and is consistent with recent examinations of the effectiveness of aCompCor (Muschelli et al., 2014). Following nuisance regression, the data were spatially smoothed (6-mm FWHM Gaussian kernel) and temporally band-pass filtered (.01–0.1 Hz).

### Data analyses

#### PCG connectivity structure

**Figure 2:**
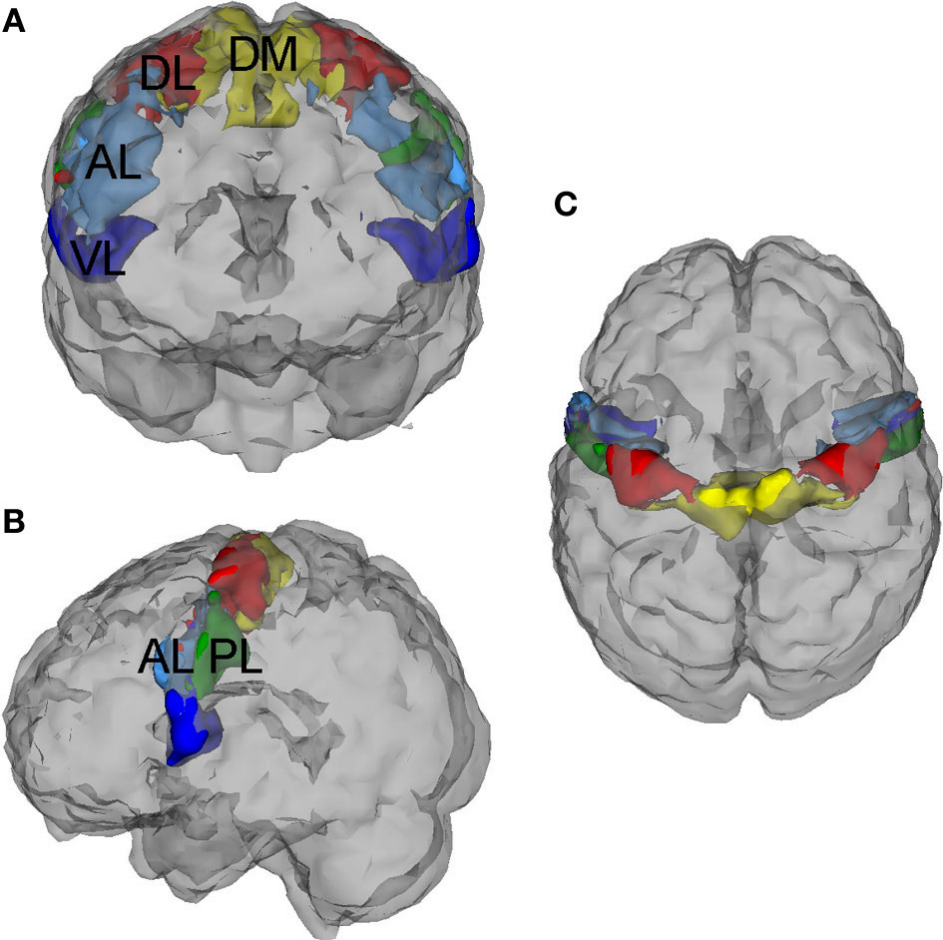
**Precentral gyrus (PCG) parcellation**. This five-region PCG parcellation was used to estimate functional motor connectivity signatures for each subject. The parcellation was derived from test-retest resting state data collected from an independent sample of 20 neurotypical adults, (Nebel et al., 2014) and the parcels were initially eroded to reduce the influence of boundary voxels on between-parcel connectivity estimates. These functional subdivisions are named based on their location within the PCG: (1) Dorsomedial (DM), (2) Dorsolateral (DL), (3) Anterior Lateral (AL), (4) Posterior Lateral (PL), and (5) Ventrolateral (VL). **(A)** Anterior (front) view, **(B)** Left side view, **(C)** Superior (birds-eye) view.

A five-region PCG parcellation was used to estimate motor FC signatures for each subject (Figure 2). The parcellation was derived from test-retest resting state data collected from an independent sample of 20 neurotypical adults and was shown to be highly reliable at the group level across imaging sessions (Nebel et al., 2014). The parcels are named based on their location within the PCG: (1) Dorsomedial (DM), (2) Dorsolateral (DL), (3) Anterior Lateral (AL), (4) Posterior Lateral (PL), and (5) Ventrolateral (VL). Both the within-parcel right-left symmetry and the overall dorsomedial to ventrolateral segregation of the parcels reflects the general configuration of the motor homunculus, suggesting that the DM parcel represents PCG resources dedicated to lower limb/trunk control while the VL parcel encompasses PCG resources reserved for the movement and coordination of the face and mouth. The parcels were initially eroded to reduce the influence of boundary voxels on between-parcel connectivity estimates. For each subject, timecourses were averaged across voxels within each eroded parcel to obtain five representative signals of PCG function. Pairwise Pearson correlations between these five representative signals were computed and converted to z-scores using the fisherz() function in R.

To evaluate the strength of connectivity within each PCG subregion, we calculated the average Pearson correlation between every pair of voxels within a subregion. The resulting 5 within-parcel connectivity scores combined with the 10 between-parcel connectivity scores represented the connectivity structure of the PCG for each subject.

#### Association between PCG connectivity structure and ASD

All analyses were implemented using the R software (R Development Core Team, 2008). We explored the association between ASD and the connectivity structure within the PCG using a logistic regression model, which included a number of covariates to account for potentially confounding sources of variability between the groups. Another potential approach to this question would have been to perform a series of 15 two-sample *t*-tests for each element in the PCG connectivity structure; however, we used the logistic regression approach so that we would be able to model the complicated interplay between functional connectivity and the other important demographic and quality control variables. We modeled between-parcel and within-parcel connectivity associations with the likelihood of belonging to the ASD group separately. For each logistic regression model, participants were coded according to their diagnosis. Suppose Y_i_ = 0 if the ith participant belongs to the control group and Y_i_ = 1 if the ith participant belongs to the ASD group. Then,

$$\text{logit}[\text{P}({\text{Y}}_{\text{i}}=1)]=\beta_{0}+X_{\text{i}}\beta_{1}$$

where β_0_ is the intercept showing the average log odds of ASD when all covariates are equal to zero, for each subject i, X_i_ is the vector of subject-specific connectivity scores (either 10 between-parcels or 5 within-parcel) and covariates added to account for potential confounders on the relationship between diagnosis and the connectivity structure. We included age, FIQ, and categorical handedness (right, left, or mixed/ambidextrous) as covariates. Two important image quality metrics were also included as covariates: (1) mean FD and (2) the spatial correlation between the first (stabilized) volume of each subject's MNI-registered data and SPM's EPI template (Allen et al., 2011). We constructed our image processing pipeline with the goal of minimizing the residual effects of motion at the individual level; however, it is still important to consider potential motion effects at the group level (Fair et al., 2013; Redcay et al., 2013; Yan et al., 2013). Similarly, with such a large sample, we thought it important to account for variability in the consistency of spatial normalization across subjects. Data collection sites were also included as factors in the model so that a coefficient was estimated for each site individually relative to the baseline site. These site coefficients attempted to account for the average difference of the outcome (odds of ASD diagnosis) for a given site relative to the baseline site, which in this case was California Institute of Technology. The exponentiated form of the log odds coefficients are reported for variables that were significant predictors of diagnosis along with the corresponding *p*-value.

#### Association between PCG connectivity structure and autism trait severity

ASD-like traits vary among non-clinical individuals, with those satisfying a clinical diagnosis of ASD falling at the extreme end of a spectrum that encompasses the population at large (Baron-Cohen et al., 2001; Constantino and Todd, 2003; Mandy and Skuse, 2008). To investigate the association between autism trait severity and PCG connectivity structure across the aggregate sample, we used a multiple regression approach. The same independent variables used in the logistic regression were included in the multiple regression but the total raw score from the Social Responsiveness Scale (SRS) was the outcome (Constantino et al., 2003). The SRS questionnaire was designed to aid clinical identification of individuals with ASD and generates a total score that serves as a social deficit severity index. This index has been shown to reliably distinguish individuals with ASD from individuals with other psychiatric disorders (Constantino and Todd, 2003) and is strongly associated with the social deficits criterion of the ADI-R (Constantino et al., 2003). In addition, total SRS scores appear to be continuously distributed in the general population and consistent across informants (e.g., parents and teachers) (Constantino and Todd, 2003; Constantino et al., 2003). The questions on the SRS probe an individual's level of motivation to engage in social interactions, his/her ability to recognize emotional and interpersonal cues from other people, to interpret those cues correctly and to respond to what he/she interprets appropriately in naturalistic settings. Higher scores indicate more severe social skill deficits. Once again, we modeled between-parcel and within-parcel connectivity associations with autism trait severity separately.

While the SRS is quick and easy to administer to both clinical and non-clinical populations, it is not considered the gold standard for assessing the symptoms of autism. The ADOS, on the other hand, is a semi-structured autism diagnostic observation and has become the common choice among phenotyping measures for autism severity (Gotham et al., 2009). However, it is both time-consuming and costly for the ADOS to be administered by an individual trained to be research reliable. As a result, the ADOS is rarely administered to control subjects. To investigate the association between autism symptom severity and PCG connectivity structure within the ASD group, we designed two additional multiple regression models with total ADOS as the outcome and the same independent variables as were used for the SRS models (one for between-parcel connectivity and one for within-parcel connectivity).

## Results

### Association between PCG connectivity structure and ASD

**Figure 3:**
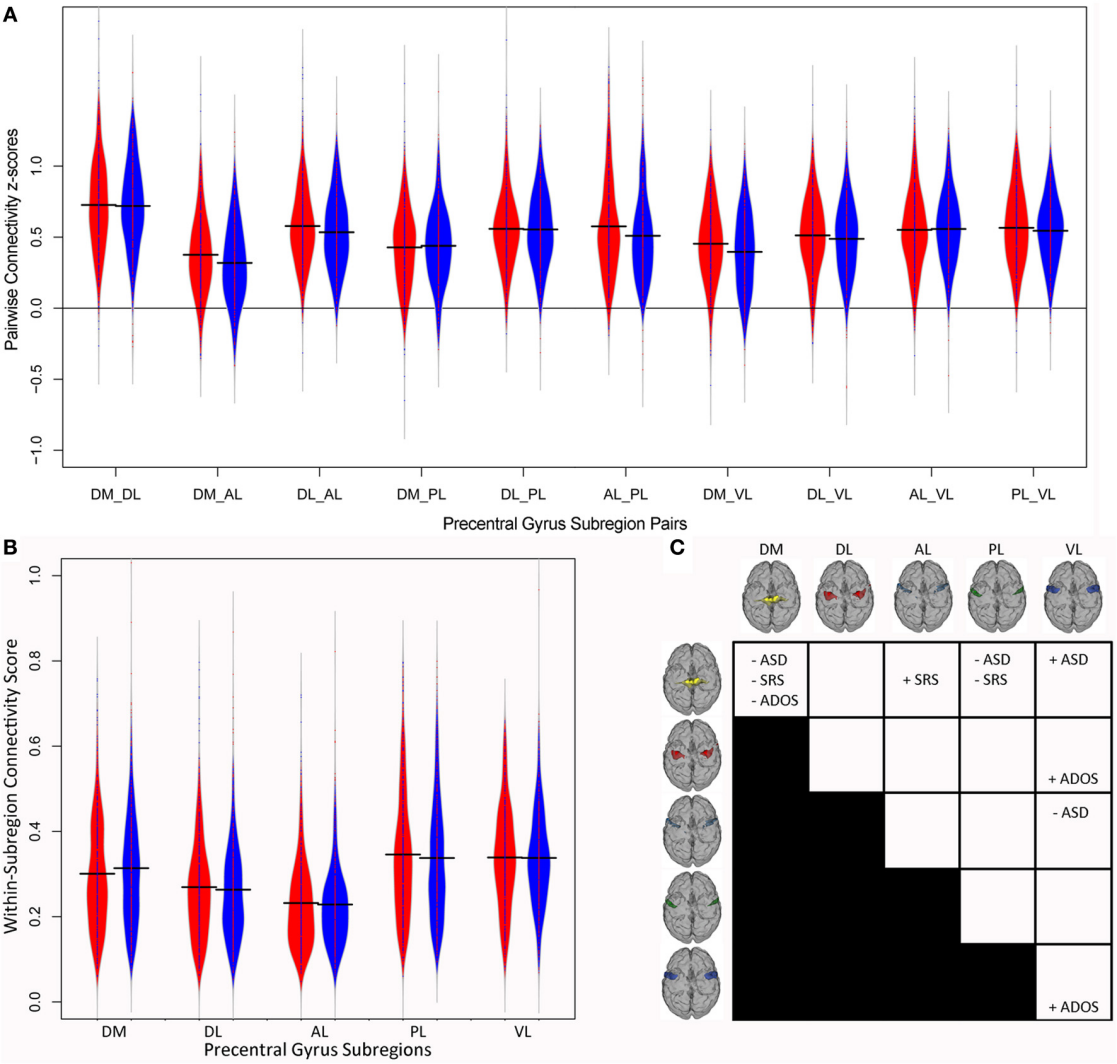
**Precentral Gyrus connectivity structure**. Violin plots of the connectivity z-scores for **(A)** the 10 pairs of precentral gyrus subregions and **(B)** within each subregion for each diagnostic group. Subjects with autism are shown in red and control subjects in blue. **(C)** Summary of associations between motor connectivity and autism. ADOS, total score on the Autism Diagnostic Observation Schedule; AL, Anterior Lateral; ASD, odds of autism; DL, Dorsolateral; DM, Dorsomedial; PL, Posterior Lateral; SRS, total Social Responsiveness Scale score; VL, Ventrolateral.

Figure 3A illustrates the distributions of between-parcel functional connectivity z-scores for the 10 pairs of PCG parcels for individuals with ASD (in red) and controls (in blue). Consistent with previous attempts to analyze multisite imaging data (Brown et al., 2012; Eloyan et al., 2012), some of the demographic covariates included in the logistic regression model influenced the diagnostic odds ratio. Although the dataset was roughly balanced for age, group differences in the distributions of FIQ and mean FD were present (Figure 1). After correcting for the effect of the other variables, a unit increase in FIQ resulted in a small but statistically significant reduction in the odds of ASD by 3% (*e*^β^ = 0.97, *p* < 0.001), while a 0.1-mm increase in mean FD resulted in a 21% increase in the odds of ASD (*e*^β^ = 7.01, *p* = 0.006). Site, age, categorical handedness, and registration quality were not significant predictors of ASD odds.

After correcting for the effect of the other variables, as outlined in the Methods, connectivity between the DM and two other PCG subregions was predictive of the odds of ASD. Consistent with our hypothesis, stronger functional coupling between the DM and VL parcels was associated with increased odds of ASD (*e*^β^ = 2.37, *p* = 0.036), indicating that individuals with ASD showed increased DM-VL coupling compared to controls. Interestingly, DM-PL connectivity was associated with decreased odds of ASD (*e*^β^ = 0.36, *p* = 0.017), indicating that individuals with ASD showed decreased DM-PL coupling compared to controls. Stronger functional connectivity between two other PCG subregions, AL-VL, was also associated with decreased odds of ASD (*e*^β^ = 0.295, *p* = 0.001), indicating that individuals with ASD showed decreased AL-VL coupling compared to controls. Functional connectivity differences involving the DM and AL regions and the rest of the brain outside of the PCG have previously been reported in children with ASD (Nebel et al., 2014); these findings suggest that the way DM and AL are connected to the other functional subregions within the PCG is also disrupted in individuals with ASD.

Figure 3B illustrates the distributions of within-parcel functional connectivity scores for each group. After correcting for the effect of the other variables, local connectivity within one PCG subregion was a significant predictor of the odds of ASD. The strength of local connectivity within the DM region was associated with decreased odds of ASD (*e*^β^ = 0.05, *p* = 0.003); in other words, a 0.1 increase in local DM connectivity resulted in a 25% reduction in the odds of belonging to the ASD group. The effects of covariates in the within-parcel model were very similar to those for the between-parcel model. FIQ was a significant predictor of ASD odds (*e*^β^ = 0.97, *p* < 0.001), as was mean FD (*e*^β^ = 21.902, *p* < 0.001).

### Association between PCG connectivity structure and autism trait severity

Because ASD-like traits vary dimensionally across the population at large, we wanted to investigate the association between ASD trait severity, as measured by total SRS, and connectivity within the PCG collapsed across both groups. Data from 358 participants (183 ASD; 175 controls) were included in the multiple regression because only select sites reported SRS scores. Similar to the logistic regression results, some of the demographic covariates influenced total SRS. Although the aggregate sample was relatively balanced for age across ASD and control groups, age was associated with autism trait severity in the smaller sample of participants with total SRS scores after correcting for the effect of the other variables included in the model; older participants demonstrated slightly more severe autistic traits (β = 1.147, *p* = 0.014). Mean FD was also associated with total SRS; subjects who moved more in the scanner displayed more severe traits (β = 58.49, *p* = 0.009), and similar to the logistic regression where higher FIQs were associated with decreased odds of ASD, participants with higher FIQs had less severe autistic traits (β = −0.579, *p* < 0.001). These associations highlight the importance of including demographic and quality metrics into the models to account for their potentially confounding effects on connectivity metrics.

After accounting for all of the other variables, functional connectivity between two pairs of PCG parcels was associated with total SRS. Once again, connectivity with the DM and AL subregions of the PCG proved to be important predictors of our outcome measure. For the logistic regression, stronger DM-PL connectivity was associated with decreased odds of ASD. Consistent with this finding, multiple regression revealed that stronger DM-PL connectivity was significantly associated with lower total SRS (β = −27.905, *p* = 0.037), so that individuals with stronger connectivity between the DM and PL parcels showed less severe social deficits. In addition, connectivity between another pair of PCG functional subunits that was only marginally associated with the odds of belonging to the ASD group (*p* = 0.059) nevertheless turned out to be associated with ASD trait severity across groups. DM-AL connectivity was positively associated with total SRS; individuals with stronger functional coupling between the DM and AL parcels demonstrated more severe social deficits (β = 32.85, *p* = 0.022).

After accounting for all of the other variables, functional connectivity within one PCG parcel was also associated with total SRS. Stronger local connectivity within the DM region was a significant predictor of lower total SRS (β = −75.992, *p* = 0.015). Just as individuals with stronger within-DM connectivity had lower odds of belonging to the ASD group, individuals with stronger local DM connectivity also displayed less severe autistic traits. Once again, both the strength of connectivity within the DM region as well as the strength of connectivity between the DM and other subregions of the PCG proved to be associated with our outcome measure. The effects of covariates in the within-parcel model were very similar to those for the between-parcel model. FIQ was a significant predictor of total SRS (β = −0.544, *p* < 0.001), as was mean FD (β = 89.2295, *p* < 0.001).

We also investigated the association between the diagnostic gold standard of ASD, total ADOS, and connectivity within the PCG using a multiple regression approach. Because the ADOS is not normally administered to controls, this analysis was restricted to individuals in the ASD group. Fifteen sites shared ADOS scores and a total of 323 participants were included in the regression. FIQ was also weakly associated with total ADOS (β = −0.031, *p* = 0.034); within the ASD group, individuals with higher FIQs demonstrated fewer autistic characteristics. Although mean FD varied between the ASD and control groups, mean FD was not significantly associated with autism severity within the ASD group. After accounting for all of the other variables, functional connectivity between the DL and VL parcels was found to be a significant predictor of total ADOS (β = 2.673, *p* = 0.043); individuals with stronger DL-VL coupling, and thus weaker segregation between these subregions, demonstrated more autistic traits. Borderline associations between total ADOS and within-parcel connectivity strength were observed for the DM and VL regions (β = −5.657, *p* = 0.059; β = 5.08, *p* = 0.047, respectively).

## Discussion

The current study found that the strength of local connectivity within PCG functional subregions and connectivity between PCG functional subregions were related to ASD diagnosis and to the severity of ASD traits (Figure 3C). Previous examination found that the precentral gyrus is organized into functional subunits reflecting distinct motor representations (Nebel et al., 2014). In that study, ASD children showed atypical organization of these subunits, with a more extensive representation of the trunk/lower limb (i.e., larger DM parcel), as well as more fractionation within premotor and multi-joint coordination regions (AL). Here, we examined the strength of connectivity within each subregion as well as the strength of functional coupling between PCG subregions in ASD individuals, using the large, multi-site ABIDE dataset. After correcting for a number of potential confounds (site, age, FIQ, categorical handedness, head movement, registration quality), connectivity within the DM parcel was associated with ASD odds; functional coupling between a number of motor subregions was also associated with ASD odds. Consistent with the hypothesis of reduced motor segregation in ASD, stronger DM-VL connectivity was associated with increased odds of ASD. In addition, weaker connectivity between DM-PL and AL-VL were associated with increased odds of ASD. These results support previous findings that motor organization is atypical in individuals with ASD (Nebel et al., 2014).

### Atypical organization of the motor cortex in ASD

The parcels used to examine connectivity within the PCG in this study reflect the general organization of the motor homunculus and were derived by clustering patterns of connectivity between PCG voxels and the brain outside of the PCG in neurotypical adults (Nebel et al., 2014). Using the same clustering approach, we previously identified differences in the size and segregation of these PCG subregions in children with ASD compared to their typically developing peers, again based solely on the patterns of connectivity between PCG voxels and the brain outside of the PCG. In this study, we wanted to further investigate whether patterns of connectivity within the PCG were abnormal in individuals with ASD using a large, independent multi-site cohort and to examine associations between PCG connectivity and diagnostic severity in this cohort. Although we previously identified group-specific PCG parcels, we chose to use the parcellation based on adult data in this study for two reasons: (1) given that our sample included children, adolescents and adults, it made sense to use the fully mature adult representation and (2) the adult parcellation was found to be highly reliable on retest (Retest data was not available for our pediatric sample).

Using these parcels, we found that functional connectivity within the DM parcel was predictive of ASD odds. Individuals with reduced functional connectivity between voxels within the DM were more likely to belong to the ASD group than to the control group. Our previous main finding based on connectivity with the rest of the brain was that the DM cluster was larger in children with ASD and encompassed much of the space occupied by the DL parcel in typically developing children and adults, suggesting that lower limb/trunk (DM) and upper limb/hand (DL) representations are less distinct in children with ASD (Nebel et al., 2014). That we observed reduced functional connectivity between voxels belonging to the DM region in individuals with ASD in the present study suggests that the functional integrity of the DM region is reduced in ASD, which may have contributed to the reduced segregation previously observed between the DM and adjacent DL parcels in children with ASD. This finding is also consistent with previous reports of decreased local functional connectivity within the precentral gyrus (Shukla et al., 2010) and reduced structural integrity of short-distance white matter fibers in children with ASD (Shukla et al., 2011).

Functional coupling between three pairs of motor subregions was also predictive of ASD diagnosis. The orofacial subregion of the PCG (VL) was more strongly connected with the lower limbs/trunk area in individuals with ASD compared to controls, and this augmented DM-VL connectivity was predictive of the odds of ASD. Truncal motor and oromotor control are established early in human development, as compared to control of the hand/fingers necessary for manual dexterity. Given this developmental trajectory, it may be that increased DM-VL connectivity in people with ASD is reflective of reported patterns of early overgrowth of localized connections in autism (for a review, see Wass, 2011) and is consistent with recent reports of short-range functional hyperconnectivity in children with ASD (Keown et al., 2013; Supekar et al., 2013).

We did not observe a significant increase in DM-DL connectivity in the ASD group in this study. Given that postural adjustments of the lower limbs and trunk are closely linked to voluntary movements of the upper limbs (Hodges et al., 1999), it is not surprising that the upper and lower limb regions of the PCG were strongly connected in both the ASD and control groups. Among all of the pairs of PCG subregions, the DM and DL were the most strongly connected. However, holding all other demographic and quality control metrics the same, individuals with ASD showed weaker DM-PL connectivity than controls. Although the PCG parcellation we used is generally consistent with the organization of the motor homunculus, the functional relevance of the elongated PL subregion is not well understood. The cortical territory labeled PL encompasses previously reported activation foci for tongue movements toward its ventral end (Alkadhi et al., 2002; Grabski et al., 2012), as well as the observation of arm/hand movements toward its dorsal end (Buccino et al., 2001; Aziz-Zadeh et al., 2006). Weaker DM-PL connectivity in ASD may thereby reflect decreased coordination of upper body and lower body movements more generally in children with autism (Jansiewicz et al., 2006; Rinehart et al., 2006; Fournier et al., 2010). Future investigation of the association between PCG connectivity and measures of body coordination in individuals with ASD is needed to confirm the functional relevance of these connectivity abnormalities.

Reduced functional connectivity between the AL and VL parcels was also associated with higher odds of an individual belonging to the ASD group. The AL subregion, near the hand knob but more anteriorly and ventrally situated, is in a region of the precentral gyrus that is consistent with ventral premotor BA6. Visual, tactile and proprioceptive information all converge in this region (Graziano, 1999) and it is also well connected with higher-level cognitive areas (Kantak et al., 2012). Activation in the AL region has been observed during observational and imitation learning (Vogt et al., 2007) and has been implicated as important for understanding the actions of others (Umiltà et al., 2001). Decreased connectivity between the AL and VL regions may thereby reflect reduced top-down control on oro-motor function in autism.

### Relationship between impaired motor control and social and communicative deficits in ASD

In addition to being predictive of the odds of ASD, the functional integrity of the DM was also predictive of the severity of autistic traits. Individuals with reduced functional coherence within the DM had higher odds of belonging to the ASD group, and they also demonstrated more severe traits as measured by total SRS score. Similarly, individuals with weaker DM-PL connectivity had higher odds of belonging to the ASD group, and they also demonstrated more severe social deficits. Although it was not predictive of the odds of ASD, connectivity between the foot (DM) and premotor (AL) subregions was also predictive of total SRS score. If there was no overlap in total SRS scores for the two diagnostic groups, we would expect the same variables to predict both ASD odds and total SRS score. However, ASD-like traits vary among non-clinical individuals, and as is illustrated in Figure 1, some overlap of social deficits exists between the two groups examined in this study. Due to this overlap, it is not surprising that a correlation that was not predictive of ASD diagnosis was associated with total SRS. In this case, individuals with stronger functional coupling between the DM and AL subunits displayed more severe social deficits. In addition to these associations with total SRS score, we also observed an association between total ADOS score and DL-VL connectivity. Within the ASD group, increased functional coupling between the DL (hand) and VL (mouth) parcels was associated with the severity of ASD symptoms as measured by ADOS total score. This mixed pattern of increased and decreased functional coupling within the PCG being related to the severity of ASD traits may be related to the wide age range of our sample and diverging functional connectivity trajectories within the groups. The findings which indicated that stronger connectivity within the PCG predicted more severe social deficits may be driven by the younger participants in the sample and reflect reported patterns of early overgrowth of localized connections in autism. The relationships indicating that reduced functional connectivity within the PCG predicted less severe social deficits may be driven by the older adolescents and adults who have had time to develop compensatory mechanisms. Examination of the trajectory of functional connectivity within the motor system and its impact on both motor and social skills in individuals with autism across their lifespan may help to create a more cohesive explanation of these mixed findings.

Abnormal connectivity between functional subregions of the motor control system may lead, not only to basic motor impairments, but also to difficulties learning and performing complex, skilled gestures. Gestures may facilitate language learning (Iverson and Goldin-Meadow, 2005) and even in neurotypical adults, gestures and speech are highly synchronous (Iverson and Fagan, 2004), suggesting that the altered functional connectivity within the PCG that we observed in individuals with ASD may affect not only basic motor skills, but also social and communicative behavior (Nebel et al., 2014). In fact, we observed associations between the strength of connectivity between functional motor subregions and ASD trait severity both across ASD and control groups and within the ASD group.

### Limitations

Several important limitations of this study warrant discussion. We set out to explore differences in functional motor connectivity in individuals with ASD compared to neurotypical children and adults. Thus we limited our statistical analyses to fMRI data in a masked region in a predefined set of PCG parcels. The motor parcellation used in this study was derived from an independent dataset by clustering voxels based on how each voxel within the precentral gyrus was connected with the rest of the brain outside of the precentral gyrus. This suggests that these subdivisions of the PCG are differentially connected with the rest of the brain, or in other words, that functional subnetworks exist within the larger motor control network. We would assume that the connectivity differences observed between subregions of the PCG would extend more generally to functional subdivisions of the entire motor network, but we did not test this directly. As a next step, we can consider using the complete connectivity matrix for the whole brain to explore whether the observed brain-behavior relationships are specific to the precentral gyrus, to the larger motor network or are distributed more generally throughout the brain.

As discussed in the Data Analyses section, a mask of functional subdivisions of the PCG based on an independent dataset from 20 neurotypical adults was used in this study. The ABIDE dataset provides a unique opportunity to obtain population level maps of motor subregions for a large group of children and adolescents with ASD. However, functional parcellations based on 5–6 min of resting state data using current clustering algorithms are considerably noisier and less reliable at the participant level than at the group level. We are actively working to improve our estimates of motor organization at the participant level, both in terms of reliability and computational complexity required to produce individual subject parcellations.

Although the ABIDE dataset provides a powerful resource for examining associations between intrinsic motor connectivity and both the odds of ASD and ASD symptom severity, the relevance of increased or decreased functional coupling between subregions of the PCG to motor behavior is still unclear. Motor skill measures for this aggregate dataset were not available, but as we continue to build up our own dataset of resting state data and motor assessments from children with ASD, we will be able to better deconstruct the behavioral relevance of within-M1 connectivity by relating it to various motor skill deficits. Relating these functional connectivity differences to the underlying structural connectivity and neurochemical composition of the PCG would also help to create a more comprehensive understanding of connectivity abnormalities associated with ASD.

Despite these limitations, we were able to identify connectivity abnormalities within and between functional subunits of the precentral gyrus that were related to ASD diagnosis and to the severity of ASD traits in a large, heterogeneous sample of individuals with ASD. In particular, the functional integrity of the lower limb/truck (DM) region of the PCG, which previously had been found to be abnormally connected with the rest of the brain, was predictive of both the odds of ASD and the severity of autistic traits. Our findings provide further support for a link between motor and social/communicative deficits experienced by individuals with ASD.

### Conflict of interest statement

The authors declare that the research was conducted in the absence of any commercial or financial relationships that could be construed as a potential conflict of interest.
